# *If it wasn’t for us*,* there would be no data*: stakeholders’ perspectives on patient involvement in the use of health data in Ireland

**DOI:** 10.1186/s40900-025-00761-9

**Published:** 2025-07-28

**Authors:** Tina Bedenik, Fiona Geaney, Barbara Foley, Rachel Flynn, Kathleen E. Bennett

**Affiliations:** 1 Data Science Centre, School of Population Health, RCSI University of Medicine and Health Sciences, Dublin, Ireland; 2 FutureNeuro Research Ireland Centre for Translational Brain Science, RCSI University of Medicine and Health Sciences, Dublin, Ireland; 3 Health Information and Standards Directorate, Health Information and Quality Authority (HIQA), Cork, Ireland

**Keywords:** Qualitative research, Health data, Patient and public involvement, Primary use, Secondary use, Purpose, Ethical use, Ireland.

## Abstract

**Background:**

Legislative reform in Ireland and Europe, including the introduction of a Health Information Bill in Ireland and the European Health Data Space (EHDS) Regulation, promote strong governance of health data, including control over how health data is used for different purposes, such as individual care, research, planning and policy-making. The aim of this study was to explore key stakeholders’ perspectives on the role of patients in enabling inclusive and ethical use of health data for primary and secondary purposes in Ireland.

**Methods:**

This was a cross-sectional qualitative study with focus group design. Thirty-five participants were evenly distributed across five groups: Academics and Researchers; Data Controllers, Data Protection Officers and Ethics Experts; Patients and Public; Healthcare Professionals and the Industry Group. A semi-structured approach guided by a topic guide was used, and thematic data analysis was conducted.

**Results:**

This study identified strong support for increased patient involvement. However, contradictions in participants’ views within and across groups were found particularly around patient control over health data and data ownership and embedding Patient and Public Involvement (PPI) in research. Most of the participants agreed that patient autonomy over health data is of ‘vital’ importance; yet they advocated for staged and delayed patient access. Similarly, the participants believed that PPI was required to drive the direction of research and funding allocation; however, patients’ lack of understanding of research areas was a challenge. Concerns were expressed around informed consent required for sharing of patient data, particularly with the industry, and around the timing of consent when patients are at their most vulnerable.

**Conclusion:**

Participants expressed strong support for increased patient involvement in the use of health data in Ireland; however, there were contrasting views in relation to data control and ownership, consent processes and PPI. These findings have implications for policy development in the implementation of the EHDS in Europe, and the establishment of the Health Data Access Body in Ireland. This study emphasises the importance of patient involvement to support successful implementation of new health information systems and data access infrastructure.

**Supplementary Information:**

The online version contains supplementary material available at 10.1186/s40900-025-00761-9.

## Abstract (plain language summary)

New legal and regulatory frameworks are being introduced across Europe, including the European Health Data Space Regulation, which will make it easier to use and exchange electronic patient health records for care in the European Union. This regulation also promotes strong control over how health data is managed and used for different purposes, including health research. Data that is used for individual health care is referred to as primary use, and if it is used for other purposes, for example in research, it is referred to as secondary use. This study aims to explore the perspectives of key people on the role of patients in how their health data is used for primary and secondary purposes in Ireland. The study involved focus groups with academics and researchers; data and ethics experts; healthcare professionals; patients and public; and the industry. Focus groups involve meetings with participants with different backgrounds to discuss specific topics to help explore different perspectives. The findings suggested strong support for increased patient involvement in decisions around how their data is used, although views were inconsistent around who should have control over, or who owns health data, and how to incorporate Patient and Public Involvement (PPI) in research. This study emphasises the importance of increased patient involvement to support successful implementation of new health information systems and policies in all European Union (EU) Member States.

**Supplementary Information:**

The online version contains supplementary material available at 10.1186/s40900-025-00761-9.

## Background

Health data is any data that is related to a person’s physical or mental health status and can include data on diagnosis (e.g. biomarker and genetic data), treatment plans, and other information, for example, from wearable devices that track health metrics [1]. As per the definitions set out in EU Regulation 2025/327 (Article 2), the primary use of health data is focused on the direct use for healthcare, while the secondary use of health data involves reusing health data for purposes other than those for which they were originally required [2, 3]. The primary use of health data enables providing real-time, direct care to the patients, as well as patient access to and sharing of their digital health data to ensure continuity of care [4]. The secondary use of health data can inform planning and management of healthcare services, guide policy making, inform health technology assessment of new technologies and identify opportunities for future research and innovation, thereby leading to improved knowledge about diseases and treatments and improve patient outcomes [2]. A digitalised health environment where data is used, for primary and secondary purpose, in an effective and secure manner is likely to encourage trust in data management systems, promote safer better care, and improve health and wellbeing outcomes for service users [2, 5]. Notwithstanding the benefits of primary and secondary use of health data, questions remain about the processes to access and share data, ownership of the data, the need for consent, and the role of the patients in how their own data is accessed, shared and used in future research [6, 7]. Although concerns relating to confidentiality, control over data, misuse of data, discrimination and stigma have been reported, patients and public are generally supportive of data use, sharing and linkage if the organisation conducting or sharing the data is considered a trusted body and there is actual or perceived benefit for the public [8–13]. In addition, previous research suggests that there is a need to support public education and information provision around the existing practices, security and safeguards related to primary and secondary use of health data, in order to address low levels of awareness among patients and public exist [6, 8–13]. For example, misunderstanding exists in relation to health data definitions. Data ownership is often confused with terms including data controller, data processor and joint controller and the rights attributed to a data subject including “right to access” and “health data holders” [14–17]. Although these terms are clearly outlined in the legislative frameworks, it is important that these terms are aligned and clearly explained within the Irish Healthcare landscape.

Since 2018, significant European legislative developments, including the General Data Protection Regulation (GDPR) recognise that high quality data can improve the efficiency of services, while recognising service users concerns regarding confidentiality, inclusivity, and ethical considerations [1, 18, 19]. Additional legislative reform in Europe and Ireland including the European Health Data Space (EHDS) Regulation and the Health Information Bill promote the re-use of data, efficient exchange of data and reinforce the requirement for strong governance of health data including sufficient safeguards for data protection, security, confidentiality and ethical use [5, 17, 20–22]. The EHDS Regulation (EU) 2025/237 became effective on 26 March 2025 and stated that the European Commission must adopt implementing acts. In March 2029, important parts of the EHDS Regulation will enter into application, and in March 2031 all EU Member States will have enabled the exchange of 17 categories of health data [17]. This comes after Regulation (EU) 2016/679 GDPR and Regulation (EU) 2022/868 Data Governance Act, but it is intended to provide extra protection for access to health data [1, 21]. In Ireland, as set out in Sect. 8(1) of the Health Act 2007, the Health Information and Quality Authority (HIQA) has responsibility for setting standards for health information, monitoring compliance against those standards and evaluating the quality of the health information [23]. HIQA make recommendations to the Minister for Health and Health Service Executive (HSE) in relation to improving the health information system, including adopting a standardised approach to the collection, use and sharing of health information [2].

A human rights-based approach may help to balance the ethical debate of individual privacy versus societal benefit for the primary and secondary use of health data [7, 24–26]. This approach is supported by FREDA, an internationally recognised framework through which human rights is considered under five principles: Fairness, Respect, Equality, Dignity and Autonomy (FREDA) [27]. When considering application in practice, a human rights-based approach ensures that service providers and organisations managing data consider those about whom it holds information as equal partners in planning, developing and monitoring information management policies and processes. This should inform impartial information management practices in which the health value created from the data must equitably benefit individuals, groups and communities [2, 28]. In addition, this framework supports greater Patient and Public Involvement (PPI), which can be defined as ‘a collaboration or partnership with patients, carers, service users, families, people with lived experience, or the public, in planning, designing, managing, conducting, dissemination and translation of research’ [29]. Reasons for including PPI in health research include the following: (1) researchers have a moral duty and patients have a right to have an input into research on their own conditions (2), a real-world lived experience improves the efficiency and value of research and (3) it increases the accountability and transparency of research and thus may help to attract more resources [30–33]. There is however limited evidence in Ireland on key stakeholders’ perspectives on the role of the patients in how their data should be accessed, shared and used. Key stakeholders are the main groups of people that either work with health data (Academics and Researchers; Data and Ethics Experts; Healthcare Professionals and the Industry Group), or that may be impacted by the use of their health data (Patients and Public). The aim of this study was to explore the perspectives of the key stakeholders on the role of patients in enabling inclusive and ethical use of health data for primary and secondary purposes in Ireland.

## Methods

### Study design

A cross-sectional qualitative study of key stakeholders’ knowledge and views on building trust and confidence in the secondary use of health data in Ireland was conducted. The Consolidated criteria for Reporting Qualitative research (COREQ) guidelines were followed in the conduct and reporting of this study [34]. Further details on the study design and methodology are found elsewhere [35, 36].

### Participants and recruitment

The sample consisted of thirty-five participants distributed across five study groups, with seven participants in each group: Academics and Researchers; Data Controllers, Data Protection Officers (DPOs) and Ethics Experts; Patients and Public; Healthcare Professionals; and Industry Group. A combination of purposive, convenience and snowballing sampling methods were deployed in recruitment. Participants for the Patients and Public group were recruited through social media and PPI Panels available to the researcher, and all other participants were recruited through professional networks and gatekeepers. The majority of participants identified as Irish (94%), represented across 50% of the counties in Ireland and two thirds were female. A detailed overview of participant characteristics is found in the earlier research article resulting from the qualitative study [36]. Participants in the Patients and Public group received an honorarium (voucher) for their time and contribution to the study.

### Data collection

The data was collected through online focus groups via the Zoom platform in 2023 utilising a semi-structured approach. Focus groups were guided by a topic guide (Additional file 1) that was informed by the literature review and consultations with relevant stakeholders including a PPI Panel in the RCSI University of Medicine and Health Sciences and HIQA. Participants were asked questions across three overarching themes regarding secondary use of health data: (1) knowledge, experiences and perspectives; (2) standards and regulation; and (3) public trust and confidence. The focus groups were audio recorded and transcribed verbatim, and all identifying markers were removed prior to analysis.

### Data analysis

NVivo 12 software was used to organise the data and facilitate the analysis. Data was analysed thematically to allow for flexibility, adaptability and production of rich data. The transcripts were scrutinised to identify recurrent patterns of meanings and generate themes, in line with the six phases of analysis employed in reflexive thematic analysis: familiarization with the data; generating initial codes; searching for themes; reviewing themes; defining and naming themes and producing the report [37, 38]. Both a deductive and inductive approach were implemented. The data were initially coded in one of the three overarching themes: (1) knowledge, experiences and perspectives; (2) standards and regulation; and (3) public trust and confidence, and the main themes and sub-themes were subsequently identified, organised and reduced in number. Under the overarching theme (2) standards and regulation, the theme of *patient involvement* had the highest number of references in the dataset, which is why it had been chosen as the focus of this article, and similarly the sub-themes have been chosen their prevalence in the dataset.

## Results

The theme of patient involvement captures three sub-themes: (1) patient control over health data and data ownership (2), informed consent to enable the use of patient data and (3) embedding Patient and Public Involvement (PPI) in research. Although the qualitative study focused on secondary use of health data, questions were asked about primary use as well, and participants sometimes referred to secondary and primary use interchangeably. Therefore, both secondary and primary use of health data are considered in this article.

### Patient control over health data and data ownership

The participants across all five groups discussed the ethical implications of giving patients full control over their health data for primary use. The academics and researchers agreed that patient autonomy is of ‘vital’ importance, yet they also advocated for a staged or delayed access to health data for primary use to protect patients from information that may be distressing to receive.

There are scenarios where maybe somebody doesn’t need to know about a particular risk. I’m thinking of one particular example in a rare condition where you can have a catastrophic bleed out that could happen at any time. And that’s it for you then, if that happens. And certainly, some people who found that out would rather they had never known. (Academics and Researchers; P1)

Academics and researchers thought that health data needs to be contextualised by healthcare professionals before patients gain full control over access. Releasing all the information could overwhelm the patients, who may not have the knowledge to understand the implications of their conditions.

With tests, whether you hold off and maybe give it a week so that healthcare professionals have had a chance to look at it, and maybe talk to the patient? Or do you release it straight away and then you cause the risk of maybe causing health care anxiety, them looking up stuff on Google, and not getting the correct support and counselling that they might need to understand it? (Academics and Researchers; P3)

The academics and researchers also suggested that patients could be provided with full control over their health data and exercise their right to *not* look at the data. Similarly, the data controllers, DPOs and ethics experts group favoured a cautious approach and stressed the necessity of allowing sufficient time and support to process the information. They reflected on patient data access practices internationally, in an effort to illustrate what staged access may look like.

I think it was Estonia, you have a locked box, I think also in Canada, and there are certain aspects of your care that you only allow certain people to see. So, mental health would be a big one. Obviously, anything around reproductive rights, like if you don’t want someone to see that you can tick that only those who are involved in your care see it. And I think giving people that level of access over their care is really important. (Data controllers, DPOs and ethics experts; P10).

Interestingly, unlike the staged approach advocated by academics and researchers wherein the health service may restrict patients’ access to health data, in this scenario it was the patients who had the power to restrict healthcare professionals’ access to their data. This in turn gives patients power over their data and ultimately care, and fosters transparency and trust. The participants in this group also highlighted that patients themselves may not want to have full access to their health data.

I suppose that in theory it sounds perfect. Of course, you should have access to all your data, and nothing about me without me, and all of that. But not everybody wants to know everything at any particular time. (Data controllers, DPOs and ethics experts; P14).

Nonetheless, the data controllers, DPOs and ethics experts acknowledged that giving patients control over their data and care is empowering, and underscored that ‘the more engaged patients – the better their outcomes’. In the healthcare professionals group differing views were expressed about data ownership. Some participants in this group believed that health data belongs to the health service, rather than the patients.

I could be 30 years out of date on this, but I think legally your data is actually the property of the health service that you’re dealing with. So, it’s not actually my data. When I want to know my blood results, I have to ask the GP practice to share them with me. If I want correspondence, I have to ask for it. It doesn’t routinely come to me. And it actually doesn’t belong to me. (Healthcare Professionals; P23).

Whilst some healthcare professionals contended that the health service owns the data that can be used for secondary purposes without patient consent, other participants in that group believed the exact opposite.

We will always treat that our patient data is the patients’ data in every decision that we make around the use of it. Now, we’d like for our patients to have access to their data in the register, that’s where we’re going down the road. But, we would always say it’s their data. (Healthcare Professionals; P27)

Given these conflicting views, the healthcare professionals concluded that data ownership is a ‘complicated’ matter and that EU GDPR, the regulation governing the protection of data in the European Union, ‘hasn’t been implemented so well’ [1, 18]. Participants in the industry group argued that full control over access to health data for primary use is not beneficial in circumstances such as genetic screening. Although patients sometimes may ‘prefer to be in the dark’, it was highlighted that they also need to be brought into the decision-making process.

I think one of the things that we always see absent in the conversations that we have with clients around utilisation of data is the actual data subject, patient themselves. And more often than not they’re not consulted in the way that they perhaps should be consulted. (Industry Group; P32)

This particular view was also echoed in the patients and public group, wherein participants asserted that they wish to have more control and the ability to decide who is accessing their data for primary or secondary purposes, and how.

We as patients have to take back our data. There is this belief that you go to a doctor or you go to a hospital, you just let them take care of you– you don’t even ask the questions. But I think we have to take back the knowledge, and we’ve to take back the power, and the data is ours. If it wasn’t for us, there would be no data. (Patients and Public; P21)

When queried further about the level of control that patients should have over the use of their data for secondary purposes, the patients and public group found it difficult to locate the fine balance.

I think that’s a hard question to quantify or to say, because yes, you want to stay informed, but would I want to know every single time my data was looked at, or my data was shared, or my data was used for a study? I don’t know, I don’t think so. (Patients and Public; P19)

It was revealed in the discussion with the patients and public group that the participants were informed about patient rights under the GDPR regarding the primary use of health data, including the right to withdraw consent, the right to erasure and the right to have the data rectified, and they also stressed that all patients need to be educated about these rights [24, 26].

### Informed consent to enable the use of patient data

The academics and researchers group stated that patient consent to enable the use of health data for secondary purposes needs to be sought on an ongoing basis. However, the participants emphasised that not all patients will have the same ability or capacity to give consent due to health inequalities.

There’s an element of equity in this as well, that people with lesser education or struggling with cognition or just plain lower health literacy are going to be less involved in this process always, which is a problem (Academics and Researchers; P8).

The data controllers, DPOs and ethics experts group expressed concerns around consent and data sharing with for-profit institutions for secondary use, which they thought was a ‘very sensitive area’ [39]. Although beneficial due to the industry’s access to ‘better, quicker, more resourced technology’ than those in universities, the patients need to be given the option to consent to their data being shared for secondary use.

I think a big one that comes up is around the commercialisation and when there’s big money involved at the outcome. Without their explicit consent, in the sense that if they know this is going to create this drug and X, Y and Z and they agree with that – fine. But sometimes in Ireland, a drug could be paraded on the back of a trial someone participated in, and they can’t get access to the drug, or the health service here isn’t funding that drug yet. I think that’s quite a challenge. (Data controllers, DPOs and ethics experts; P9)

The data controllers, DPOs and ethics experts believed that given the speed with which cancer research and personalised medicine is progressing, the collaboration with the industry is needed, upon the principle of transparency. In a similar vein, the industry group focused on the grey area around patient consent that occurs when data is transferred on to the private entities for secondary use.

There is definitely aggregated data sets that we’ve come across and the question is ‘Well, we don’t have explicit consent for exactly what we’re doing here, but it’s aggregated data so protection of the patient is probably there’. These patients aren’t going to be exposed, but realistically is it ethical that we use this aggregated data for whatever purpose? (Industry Group; P33)

Participants in the industry group called for a transfer of power onto gatekeepers that could make impartial decisions around consent and safeguard patients’ rights regarding secondary use of their data. Healthcare professionals on the other hand expressed concerns around high opt-out rates among patients, and how these may impact on sample representativeness when using data for secondary purposes to improve health system efficiency.

There will always be some people who find any data use sensitive and inappropriate and would opt out if given the opportunity. But the moment patients opt out, the whole dataset becomes unrepresentative. And you can’t make health service decisions if you’re going to get high opt-out rates. (Healthcare Professionals; P24)

Some healthcare professionals suggested that once all identifying information is removed, patient consent for secondary use is redundant. The patients and public group underscored that consent for secondary use is often sought when patients are at their most vulnerable, and attention needs to be given to the timing of consent processes.

Often you might only get [a consent form] on the day of the surgery, if you are very lucky it’s maybe a couple of days beforehand on the confirmation letter or email. You are already at a heightened state of stress. Are you fully aware of what you are consenting to? (Patients and Public; 21)

In addition, the patients and public considered the role of health literacy that may hinder patients’ ability to opt in and share their health data for research studies, with implications for sample representativeness and ultimately usefulness of research.

The NALA [National Adult Literacy Agency] basic language should be used… People don’t have access, not everyone has access to emails. I think an awful lot of people could be missing out, and if you are getting the data there’s no point – 95% [of data will come from] white female who has been in a private setting all their lives. (Patients and Public; P19).

### Embedding patient and public involvement (PPI) in research

The academics and researchers group contended that PPI is ‘a moving phenomenon’, and integration of PPI in health and medical research is necessary to drive the direction of research and funding allocation. This included research that is based on secondary use of health data. However, they noted that patients’ lack of understanding and knowledge on a specific research topic may preclude meaningful involvement.

There’s a significant amount of work happening at [the EU] level in relation to clinical trials, education programs for patient representatives. So while it’s a slow burn, it’s happening in the background. That being said, I’ve been on a couple of committees and the patient representatives didn’t really understand a lot of what was being said. (Academics and Researchers; P7)

Similarly, the data controllers, DPOs and ethics experts group stressed the need for building partnerships with patients and sharing control over research projects. Some participants argued that academia and the health sector have a reputation of being ‘quite paternalistic’, which needs to be challenged.

People often from a very good place are saying ‘Well, we’re doing this in the public interest or the interest of all’, but to an individual it doesn’t mean much. So actually, properly engaging with people, and giving them options [is required]. (Data controllers, DPOs and ethics experts; P10)

The healthcare professionals stated that patients can advise on consent forms and patient information leaflets, audit submissions and ethics applications – patient involvement is the ‘key’.

Having their support often brings a lot more gravitas to whatever group you’re submitting it [to]. I know that’s been very key for us – having our patients being part of that group. We often get them over the line a lot quicker than the audits or studies that we don’t have them involved [in]. (Healthcare Professionals; P27)

The healthcare professionals also emphasised the need for appropriate PPI acknowledgement and remuneration to increase representation and to assist in building trusting partnerships. Institutions in Ireland have differing policies regarding reimbursement of PPI contributors, but this activity often takes place without reimbursement, which is a moral, ethical and practical issue.

Yes, we have to make some great strides in terms of PPI, but it’s still all done on a voluntary and an altruistic sense. And that seems wrong. I don’t go to work for fun. I’m not being asked to do an extra day to support the data. But yet we expect patients and public involvement to be done in that manner, and that seems wrong to me. (Healthcare Professionals; P26)

The industry group reflected on the knowledge discrepancies and costs associated with embedding PPI in clinical trials. However, the industry participants agreed that PPI contributes towards reducing ‘tension between what is being done and what is in the public’s best interest’. For example, patient collaborators in clinical trials can identify scheduling problems that might lead to high participant dropout rates in trials.

We did [a trial] and I can’t remember the indication, but it was for children. And they interviewed the parents, and the parents looked at the schedule of assessment and said: ‘There’s no way we’d get our child to do that – you’re talking about taking blood draws first thing in the morning, there’s no way that’s going to happen.’ (Industry Group; P30).

Interestingly, the patients and public group also expressed a wish to collaborate more closely with industry, as financial resources can help to support patient organisations and therefore proliferation of PPI more widely.

There’s a huge aspect where pharmaceuticals could support patient organisations better and help fund different aspects. Because there’s lots of things that the patient organisations need support with, but don’t have the funds to be able to build education programmes, or literacy brochures, or other ways to support research that they may want to get engaged in. (Patients and Public; P21)

## Discussion

### Overview of the findings

This paper explored the key stakeholders’ perspectives on the role of patients in facilitating inclusive and ethical use of health data for primary and secondary purposes in Ireland. We found inconsistencies and contradictions in participants’ views within and particularly across groups regarding patient control over health data, consent processes to facilitate the use of patient data, and embedding PPI in research.

### Patient control over health data and data ownership

There was an agreement among the academics and researchers, data controllers, DPOs and ethics experts, and the industry that a staged or delayed patient access to their health records is required to protect patients’ well-being, despite the opinion that patient control over health data is of ‘vital’ importance and empowering. However, views were also expressed in the data controllers, DPOs and ethics experts group in support of giving patients the power to restrict healthcare professionals’ access to their health data, particularly sensitive data related to sexual and reproductive health. In contrast to these three groups, patients and public group wanted more power over health data access and use; however, they found it challenging to identify an ‘optimal’ level of control. In the healthcare professionals group conflicting views were expressed about data ownership, revealing concerning knowledge gaps with regards to GDPR and data protection.

### Informed consent to enable the use of patient data

Different perspectives between groups were identified regarding informed consent to enable the use of patient data. The academics and researchers were concerned by the varying levels of health literacy among patients that can impact on the willingness to provide consent, whereas the healthcare professionals focused on the high opt-out rates among patients that may influence sample representativeness. On the other hand, the data controllers, DPOs and ethics experts, and the industry group were cognisant of the ‘grey area’ that included sharing patient data with for-profit entities without explicit consent. Both groups emphasised that patients need to be given the option to consent to their data being shared, and the industry group suggested deployment of gatekeepers to protect patients’ interests. This suggests that the industry group was open to an exploration of ethical challenges that arise when monetary incentives are involved, and they were mindful and supportive of patient data protection. The patient and public were concerned with the timing of seeking consent for the use of health data for secondary purposes. This may happen as late as on the day of the surgery, when patient may be under stress, and may not be fully aware what they are being asked or are agreeing to.

### Embedding patient and public involvement (PPI) in research

Inconsistencies were also evident in participants’ views on embedding PPI in research. Notwithstanding a strong agreement across all groups that PPI was necessary to drive the direction of research and funding allocation, participants identified a number of issues that preclude PPI. The academics and researchers and the industry group centred on patients’ lack of knowledge to make a meaningful contribution, exacerbated by the cost implementing PPI in research. On the other hand, concerns around a lack of remuneration for PPI contributors were expressed, interestingly, by healthcare professionals. An absence of standardised remuneration is an ethical and practical problem that interferes with patient representation and building trust, and healthcare professionals highlighting this subject shows that participants were taking into account other groups’ interests. Finally, patients and public voiced a desire to collaborate more closely with the industry, and avail of the financial resources that can support patient organisations.

### Comparison with other research

Our findings and those of others recognise that patients have an essential role in the primary and secondary use of health data, and this is being recognised more in research, policy and legislation [12, 13, 40, 41]. However, a review by Hutchings et al. reported that although there is widespread support for health data sharing, concerns remain among patients and the public regarding privacy, data control, consent and access [13]. As identified in our study, a one size fits all approach is not sufficient to manage these concerns. Baines et al. suggest that flexible models of data control and consent would facilitate transparency to support sustained patient involvement and empowerment, and also provide an opportunity to incorporate future technological, regulatory and legal developments in health data that prioritise individual or community benefits over commercial gain [42, 43]. Davey et al. examined the knowledge and expectations of patients and doctors in Ireland regarding GDPR and future research, and identified differences in perceptions of GDPR and willingness to consent to data being used for research [44]. Our study supports their findings on the knowledge gaps among healthcare professionals regarding GDPR and data protection, and underscores the importance of appropriate and ongoing education and training for this group. In regards to how PPI should be involved in research, there is a consensus regarding a need to consider the principles of meaningful PPI in research, and include these principles to inform evidence-based guidance and frameworks that stakeholders can adapt and use within their own research to achieve this [32, 41]. Factors such as incentives, compensation and equitable benefits for PPI involvement have been previously cited, and our findings suggest these as important and influential in facilitating PPI contributions and data sharing intentions [43, 45]. There are many frameworks for supporting, evaluating and reporting patient and public involvement in research, but Greenhalgh et al. suggest that evidence-based resources that stakeholders can use to co‐design their own frameworks may be most useful [32].

Our previously published article from this qualitative study explored the challenges of secondary use of health data in Ireland, and reported on the difficulties with consent processes and low health data literacy among the patients and public and how these influence data-sharing practices [36]. The increasing complexity of consent forms and patient information leaflets, further exacerbated by socio-economic disparities also contributed. This supports the requirement for increased patient involvement in consent processes. We previously reported that the healthcare practitioners had questioned whether patients understand the concept of secondary use, and some participants in the patients and public group were unaware that their data was already used for secondary purposes. In contrast to those findings, in the present study we report that patients can also be very informed about their rights under GDPR as data holders – sometimes even more informed than the healthcare professionals. Lastly, we previously reported how patients and public with caring responsibilities encounter barriers with gaining access to health data of those they care for.

### Study implications and contributions

#### Implementation of the EHDS regulation in the EU

The study provides useful insights for key stakeholders, particularly across Europe, who use health data to inform future research, industry, policy and legislative developments and those responsible for the implementation of the EHDS Regulation. It is also of relevance to those organisations and/or bodies setting and adopting standards for health information in Europe. In Europe, the EHDS Regulation requires organisations and services to adhere to the international best practice guidelines to manage information and comply with requirements in data security, data quality and data interoperability standards. All organisations and services responsible for the implementation of the EHDS Regulation, and associated standards for health information in Europe, need to take a strategic approach to information management to ensure data is collected, used, shared, controlled and processed effectively with the appropriate safeguards to protect privacy and confidentiality. These standards must also consider using a human rights-based approach including involvement of service-users, patients, public and professionals, and incorporation of their perspectives into how health information is managed [2, 28].

#### Fostering an active patient role in data use and re-use

Patients should have a key role in the primary and secondary use of their health data, including the right to access, modify, and control their data. They should be informed about how their data is being used, include the ability to consent or opt-out, and be involved in the decision-making processes regarding data sharing [46]. The experts and policymakers should actively involve the patient perspective in the discussion and development of policies related to primary and secondary use of health data. This can be achieved through various methods, including patient advisory boards, focus groups and surveys, to ensure that patient perspectives are considered in the design and implementation of data-related policies and practices. Some participants in this study advocated for a staged patient access to health data for primary use, in order to protect patients from information that may be distressing to receive. Contrary to this view, others thought that staged access could refer to patients’ right to restrict healthcare professionals’ access to their data. Interestingly, the Irish Health Information Bill 2024 may allow for both occurrences. The Bill proposes that a health services provider may restrict patients’ access to their Digital Health Record upon reasonable grounds for believing that it would likely cause serious harm to their physical or mental health (Article 12(4)) [5]. At the same time, patients may restrict access by health services providers to all or part of the patient’s Digital Health Record (Article 13(1)) [5]. However, even if patients exercise their right to restrict access, health services provider may access patient’s data when such access is necessary to protect the vital interests of the patient (Article 13(4)) [5]. This suggests that the power ultimately rests with the health services provider, and an on-going and open dialogue with patients, patient organisations and public is required to foster and support an active and engaged patient role. Information and understanding of health data and how and why it might be used is essential for this dialogue, similar to initiatives such as the UK ‘Understanding patient data’. Meaningful public and patient involvement requires provision of information on legislation such as data protection, health information and the EHDS, so that the implications of primary and secondary use of health data are fully understood. Other aspects for consideration include the use of aggregated health data, recommendations for Trusted Health Research Environments and consent for anonymisation of health data for secondary use providing exemption from data protection principles under the EU GDPR [17, 18].

#### Development of national health policy standards and recommendations

These findings also support the development of national health policy and standards for health information in Ireland, and the development of guidance and tools to help services and organisations implement these standards. The findings can be used to inform the implementation of a Health Data Access Body (HDAB), a service allowing data users, including policy makers and researchers, to apply for access to health data, within strict protocols and processing environments (required under the EHDS Regulation). The study also provides evidence in the development of recommendations to inform national policy on health information in Ireland. In collaboration with the Health Services Executive, Department of Health, Health Research Board and other key stakeholders across the Irish health system, HIQA are co-ordinating key programmes of work under the HealthData@IE project to ensure that Ireland meets its obligations relating to the secondary use of data under the EHDS Regulation. This research contributes to the body of evidence that will be used to influence the development of the Health Information Bill [5] and it will also be used to influence the implementation of the Department of Health’s ‘Digital for Care: A Digital Health Framework for Ireland 2024–2030’ which sets out a roadmap to digitally transform health services in Ireland and improve access for patients [47].

### Strengths and limitations of the study

The study has a number of strengths including the participation of a wide range of stakeholders, from diverse backgrounds and perspectives. The qualitative methodology used and data collection provided more detailed information to explore patient involvement in decisions around their own data. The study was conducted at a time when Ireland was transitioning to implementation of the EHDS and advancement of national health information legislation and rollout of the Individual Health Identifier (IHI). The findings emphasise the importance of patient and public involvement to support successful implementation of innovation and developments in the area of health information, especially in relation to the EHDS. This will be revolutionary for patients and the public, healthcare professionals, policy and decision makers.

Limitations of the study include a lack of male participation in the public and patients focus group. The method of recruitment involved purposive and convenience sampling, and use of social media, which may have introduced bias in those participants agreeing to attend the focus groups. Patient and public participants were provided with additional optional reading on secondary use of health data before the focus groups, but they were not asked explicitly if they had read these, so it is unclear whether this would have had any impact. Another limitation was that different experts, for example ethical and technical, were combined into one group, and this may have resulted in differing viewpoints, and/or less time on the individual ethical and technical discussion. Also, expertise from specific representatives, for example, policy makers, civil servants, and governmental departments were not included and this would have contributed an additional perspective, for example, in relation to the code of conduct for civil servants and the principle of impartiality which may be of relevance [48]. Types of health data include electronic health records, administrative or claims data, patient or disease registries, health surveys, clinical trial data and genomic data. These data provide information on diagnosis, including genetic data, or treatment, which may have an impact if shared outside of direct healthcare (e.g. insurance organisations). We did not ask participants about their understanding of different types of health data and their implications. Although the findings may not be generalisable to all countries, there were aspects, including public and patient involvement and control of health data, consent processes, sharing of data with for-profit organisations and improving health literacy, which will be of interest to the EU member states also implementing the EHDS.

### Future research

Further research needs to consider the implementation of standardised reimbursement of PPI contributors to ensure that different patient voices are represented in research. In addition, the timing of consent processes requires a deeper explanation, for seeking consent for secondary use of data shortly before medical procedures impacts on patients’ ability to consent. The mechanisms for ensuring responsible sharing of health data with for-profit sector requires consideration, and the ways in which trusting partnerships can be developed between the industry and patient organisations. Finally, the rights and responsibilities of carers in the context of both primary and secondary use of health data require further consideration.

## Conclusion

The study findings provide important evidence for key stakeholders on the primary and secondary use of health data to inform future research, industry, policy and legislative developments and in the implementation of the EHDS Regulation and standards for health information in Europe and in Ireland. Patient and public involvement is essential in driving forward the use of primary use of health data to streamline healthcare, and secondary use of health data to drive research, policy, legislation and planning.

## Electronic supplementary material

Below is the link to the electronic supplementary material.

Supplementary Material 1

## Data Availability

Data generated or analysed for this study are included in this published article.

## Abbreviations

HIQA: Health Information and Quality Authority
PPI: Patient and Public Involvement
EHDS: European Health Data Space
EU: European Union
GDPR: General Data Protection Regulation
DPO: Data Protection Officer
IHI: Individual Health Identifier
HDAB: Health Data Access Body
