# Supplementary material for: If it wasn’t for us, there would be no data: stakeholders’ perspectives on patient involvement in the use of health data in Ireland
Source: Res Involv Engagem. 2025 Jul 28;11:87. doi: 10.1186/s40900-025-00761-9 (PMC12306090; doi:10.1186/s40900-025-00761-9)
Supplement: Supplementary file 1 — Supplementary Material 1 [file 40900_2025_761_MOESM1_ESM.docx]

**Additional file 1**

Topic guide

Qualitative study exploring how to build public trust and confidence in the use of health data for healthcare improvement and research; Ireland; 2022-24

| **Introduction**  Introductions and housekeeping |
| --- |
| **Knowledge, experiences and attitudes**  Have you had any experiences with secondary use of health data?  What is your understanding of secondary health data use?  What do you think are the benefits of secondary health data use?  What do you consider the main risks of secondary health data use?  Who should be able to access and use health data for secondary purposes?  PROMPT: What is your opinion on sharing data with the private sector? |
| **Regulation, ethical challenges and power-sharing responsibilities**  What do you think should be the standards regarding safe use of health data?  When it is not ethical to use health data for secondary analysis?  PROMPT: Are there any types of health data that should not be shared?  Are there any social groups that may not benefit from easy access to their health data?  How much power should patients have over access to their health data?  PROMPT: What are the pros and cons of patients having full access to their health record? |
| **Trust and confidence**  How would you define trust?  PROMPT: What do you think are the key features of trust?  What makes an organisation trustworthy?  What are the actions and activities that inspire public trust?  What are the biggest obstacles to building public trust?  What is the role of patients and public in the process of building trust? |
| **Conclusion**  Is there anything else you would like to discuss that we have not explored yet? |
